# Free Training in Midwifery

**Published:** 1888-03-31

**Authors:** 


### FREE TRAINING IN MIDWIFERY.

In reply to Nurse Dore's question about free training in midwifery, Nurse Dore could not do better than apply to the Matron, at the Kensington Infirmary, Marloes Road, S.W.

Fulham. Nurse Edith.

In answer to Nurse Dore's question regarding free training in midwifery, such can be obtained from the Workhouse Infirmary Nursing Association. Nurses are bound to work for the Association for three years after. Application to the Hon. Secretary, 6, Adam Street, W.C.

